# The problem with skeletal muscle series elasticity

**DOI:** 10.1186/s42490-019-0031-y

**Published:** 2019-12-03

**Authors:** Walter Herzog

**Affiliations:** Faculty of Kinesiology, Human Performance Lab, University of Calgary, Calgary, T2N-1N4 Canada

**Keywords:** Muscle elasticity, Tendon, Aponeurosis, Muscle stiffness, Storage of energy, Release of energy, Stretch-shortening cycle, Hill model, Parallel elastic element, Muscle energetics

## Abstract

Muscles contain contractile and (visco-) elastic passive components. At the latest since Hill’s classic works in the 1930s, it has been known that these elastic components affect the length and rate of change in length of the contractile component, and thus the active force capability of dynamically working muscles. In an attempt to elucidate functional properties of these muscle elastic components, scientists have introduced the notion of “series” and “parallel” elasticity. Unfortunately, this has led to much confusion and erroneous interpretations of results when the mechanical definitions of parallel and series elasticity were violated.

In this review, I will focus on muscle series elasticity, by first providing the mechanical definition for series elasticity, and then provide theoretical and experimental examples of the concept of series elasticity. Of particular importance is the treatment of aponeuroses. Aponeuroses are not in series with the tendon of a muscle nor the muscle’s contractile elements. The implicit and explicit treatment of aponeuroses as series elastic elements in muscle has led to incorrect conclusions about aponeuroses stiffness and Young’s modulus, and has contributed to vast overestimations of the storage and release of mechanical energy in cyclic muscle contractions.

Series elasticity is a defined mechanical concept that needs to be treated carefully when applied to skeletal muscle mechanics. Measuring aponeuroses mechanical properties in a muscle, and its possible contribution to the storage and release of mechanical energy is not trivial, and to my best knowledge, has not been (correctly) done yet.

## Background

**Fig. 1:**
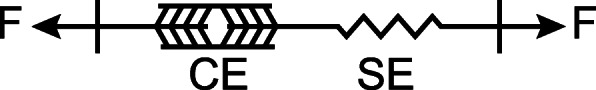
Hill model. Hill (1938) [1] proposed that muscle consists of two basic elements: a contractile force producing element (CE), and an “elastic” element that is arranged “in series” (SE) with the contractile element. Hill (1938) [1] pointed out correctly that the series elastic element influences the contractile element’s length and rate of change in length (velocity) during dynamic contractions, and thus, affects the force producing capability of the contractile element

At the latest since Hill’s (1938) [1] classic work on the heat of shortening in frog skeletal muscles, we know that elasticity and muscle elastic components play a crucial role in the mechanics of muscle contraction. Hill (1938) [1] derived a model of skeletal muscle that had a contractile element in series with an elastic element (Fig 1). The terms “in series” and “elastic” refer to the idea that the length of this element was instantaneously proportional to the muscle force. Hill (1938) [1] pointed out correctly that the amount of shortening, and the shortening speed of the contractile component was crucially dependent on the properties of the series elastic element. However, where this series elastic element was located, and what it consisted of, was not defined. On occasions, Hill (1938, 1] referred to the series elastic element as the tendon, and this is repeated in his later works (e.g., Hill, 1950) [2], but Hill acknowledged that muscle (series and non-series) elasticity might also reside in components other than the tendons.

**Fig. 2:**
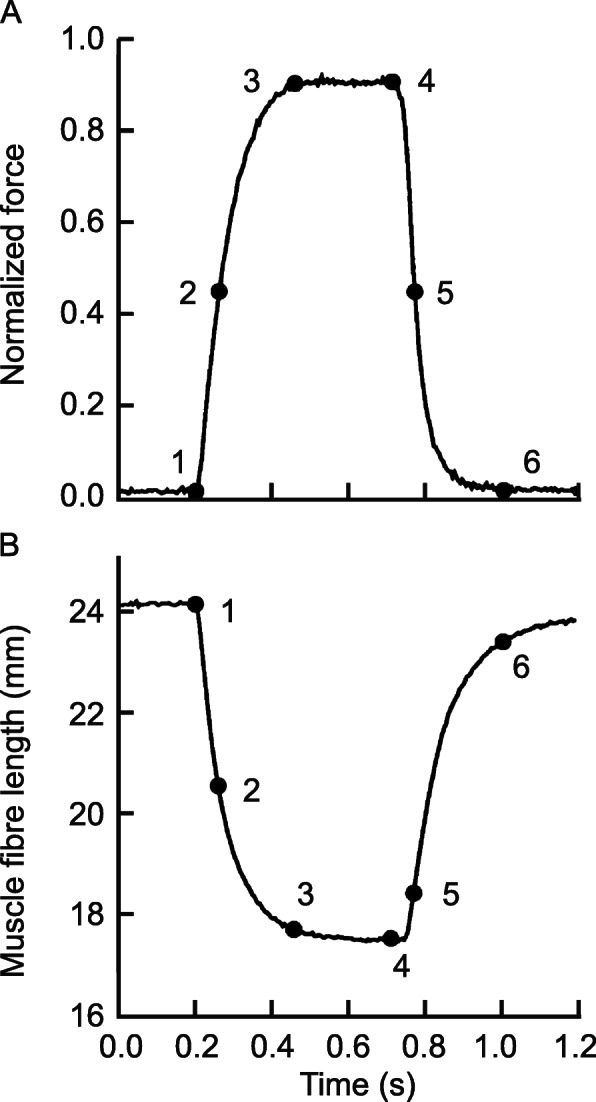
Fibre shortening. Force (**a**), and corresponding fascicle length change (**b**) for an isometric contraction of the cat medial gastrocnemius (MG) muscle. Isometric here refers to the constant length of the entire muscle-tendon unit. For this particular example, the MG muscle fascicles/fibres shorten from about 24 mm to about 18 mm with increasing force, demonstrating that fascicle/fibre/sarcomere lengths in a muscle do not depend on muscle length alone (the muscle was kept at a constant length), but also depend crucially on the amount of force that the muscle is producing. The interpretation of this finding has been that with increasing force, structural (visco-) elastic elements of the muscle are stretched, allowing muscle fascicles/fibres to shorten. The amount of shortening of muscle fibres depends on the initial muscle length and the force produced (e.g. [4–6]) [Reprinted with permission from The Physiological Society, the Journal of Physiology, Griffiths et al. 1991 [3]]

**Fig. 3:**
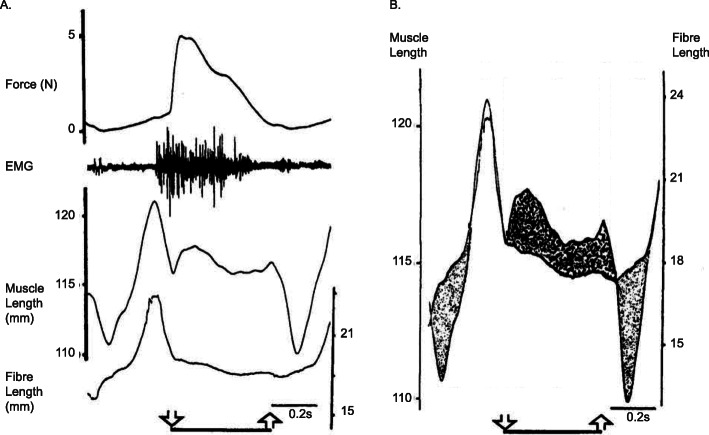
Muscle mechanics during cat walking. Force, electromyographical (EMG) signal, muscle length and fascicle/fibre length (**a**) for the medial gastrocnemius of a cat during a step cycle. The downward and upward arrows indicate paw contact and paw liftoff at the beginning and end of the stance phase, respectively. **b** Difference between muscle length changes and fascicle/fibre length changes. Note specifically that at initial paw contact, fascicle/fibre lengths decrease while the muscle is stretched, while just after paw liftoff, the opposite is correct: the muscle shortens while the fascicles/fibres are elongating. Hoffer et al. (1989) [7] were the pioneers in measuring fascicle and muscle lengths in freely moving animals simultaneously, demonstrating the importance of muscle elasticity and reinforcing the notion that muscle fascicle/fibre length did not only depend on muscle length exclusively, but also depended crucially on muscle force. [Reprinted with permission from Elsevier Science Publishers, in Progress in Brain Research, Hoffer et al. 1989 [7]]

Despite this early account of muscle elasticity, and the recognition of the substantial effects it had on the mechanics of muscle contraction, muscle physiologists and mechanists largely ignored the effects of muscle elasticity for the better part of the next half century. This state of affairs changed for good in the late 1980s, when research in the Hoffer lab, using the newly developed sonomicrometry technique, showed unequivocally that muscle fibres can shorten substantially (up to 28% with the muscle at optimal length) in an “isometric” contraction [3] (isometric here refers to the idea that the entire muscle-tendon unit length was kept at a constant length – Fig. 2), and that in the walking cat, medial gastrocnemius (MG) muscle-tendon unit lengthening was associated with MG fibre shortening, and vice versa, for distinct phases of the cat step cycle [7] (Fig. 3). This uncoupling between muscle and fascicle length changes has been observed in dozens of preparations in the meantime for isometric (e.g. [4, 5, 8, 9]), and for dynamic in vivo human muscle contractions (e.g. [10–14]).

With muscle elasticity being established as an important part of muscle function, research in this field exploded. Specifically, the role of “muscle series elasticity” in enhancing performance (e.g., [15]), reducing metabolic cost of movement (e.g. [16, 17]), and storing and releasing of elastic energy in cyclic movements (e.g., [18–22]) became prominent. However, what constitutes series elasticity in a muscle, how it is defined, and what conclusions can be drawn from studies dealing with muscle series elasticity, remains confusing for a variety of reasons. Maybe foremost, series elasticity seems to have an anatomical/structural meaning for some, and a mechanical meaning for others. It is at this intersection between anatomy/structure and mechanics where confusion has arisen that has led to misinterpretations of the mechanics of muscle contraction ([23, 24]), specifically, errors in the calculation of series elastic stiffness, Young’s moduli, and storage and release of mechanical energy. Here, we will attempt to address some of the confusion by defining series elasticity in a mechanically consistent manner, and pointing out the difficulties when interpreting series elasticity from a structural point of view and inferring mechanical properties.

## Main text

### Series elasticity

**Fig. 4:**
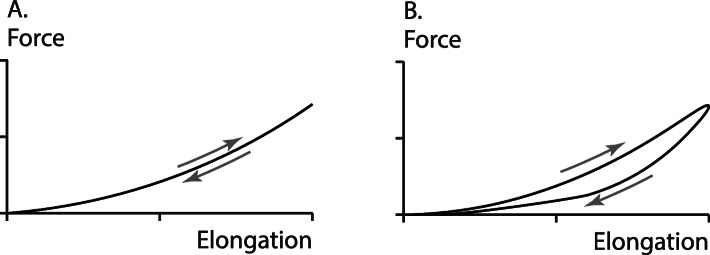
Elasticity. Force as a function of elongation in a perfectly elastic material (**a**) and in a visco-elastic material (**b**). For the elastic material, force (elongation) is uniquely given by the elongation (force), and the loading and unloading curves overlap, while the force (elongation) curve of the viscoelastic material depends on the rate of stretching/shortening and/or the rate of force application/decay. In contrast to the elastic material, where the loading and unloading energies are the same, in a visco-elastic material some of the energy supplied during stretching is dissipated, thus the energy during the unloading phase is smaller than that obtained in the loading phase, resulting in a characteristic hysteresis, as shown in (**b**)

The concept of “series elasticity” is used in structural mechanics to describe ideal situations with the aim to understand the behaviour and properties of complex systems. When two elements are said to be arranged “in series”, it implies that the instantaneous internal forces in the two elements are always the same, or at least in constant proportion, independent of the loading history and independent of the material properties. For example, in Fig. (1), the force exerted in the idealized contractile element (CE), is always matched instantaneously by the elastic spring in series with CE, the series elastic element (SE). The term “elastic” implies that the strain is instantaneously given by the force applied to the SE element. Therefore, an elastic material has the same strain for a given force, independent of the history of force application (fast or slow; or increasing vs. decreasing force (Fig. 4a). The best known example of an elastic element is the idealized, linear spring, or Hooke’s law, where the elongation of the spring is always (and instantaneously) given by the force applied to the spring; i.e. *F = kx,* where *F* is the applied force, *k* the spring constant (stiffness), and *x* the deformation of the spring from its zero-strain, unloaded length.

In the natural world, there is no perfectly elastic element. Quartz fibres are found to approximate perfect elasticity the best. Rubber is also almost perfectly elastic while tendons are not. Tendons become stiffer when forces are applied faster, thereby exhibiting visco-elastic properties, and elongation is not given by the force exclusively. Tendons also have a distinct hysteresis of about 10%, as illustrated conceptually (but not in magnitude) in the example shown in Fig. (4B), which means that the energy applied in stretching a tendon exceeds the energy that is returned by the tendon by about 10% when force is removed. A perfectly elastic material, by definition, does not have a hysteresis, as its deformation is always the same for a given force and independent of the history of force application.

One might be tempted to stop any discussion on muscle series elasticity here, as perfectly elastic materials do not exist in nature, and materials often implicated to be elastic in muscles, such as tendons, cross-bridges, titin, and aponeuroses, are not elastic ([25–27]). However, in practice, it is sometimes useful, and is done frequently in biomechanics, to consider nearly elastic materials as elastic in order to gain an understanding of complex systems. So we will proceed.

#### Series elasticity in muscles

Muscles have a number of passive (non-contractile) “elastic” elements that have been implicated with series elasticity. Some elastic elements implicated in being “in series” with the “contractile element” are the free tendon, the muscle internal aponeuroses, the structural protein “titin”, the elastic elements in the cross-bridges (the S2 element in Huxley’s 1969 [28] notation, or the AB element in Huxley and Simmons’ 1971 [29] notation), and the Z-bands in sarcomeres. Here, I will focus primarily on series elasticity of the entire muscle. However, for completeness, I will also briefly discuss cross-bridge, titin, and Z-band elasticity, as they have been implicated as being in series with some molecular or sub-cellular component of muscle.

Briefly, cross-bridge elasticity, according to classic cross-bridge models, is in series with the cross-bridge head; that is, whatever force is transmitted from the cross-bridge head to the actin filament is thought to be transmitted by an elastic element that attaches the cross-bridge head to the myosin filament backbone (S2 in Huxley’s 1969 [28] notation). In fact, in the original cross-bridge theory (Huxley, 1957 [30]), the cross-bridge head is attached to the myosin backbone via a linearly elastic spring that is arranged in series with the cross-bridge head. The force of the cross-bridge was then assumed to be given by the elongation of that linear spring from its equilibrium position: *F*_*cb*_ *= k*_*cb*_*x*; where *F*_*cb*_ is the force in a cross-bridge, *k*_*cb*_ is the (constant) cross-bridge spring stiffness, and *x* is the elongation of the cross-bridge spring element from its equilibrium position. The idea of a linear cross-bridge stiffness has been challenged [31] and is likely not correct. Nevertheless, the notion of linear elasticity in cross-bridges continues to persist. Furthermore, myofibrils and fibres of a muscle have complex (parallel) connections, and thus, cross-bridges in different myofibrils and fibres cannot be considered mechanically “in series” with each other.

**Fig. 5:**
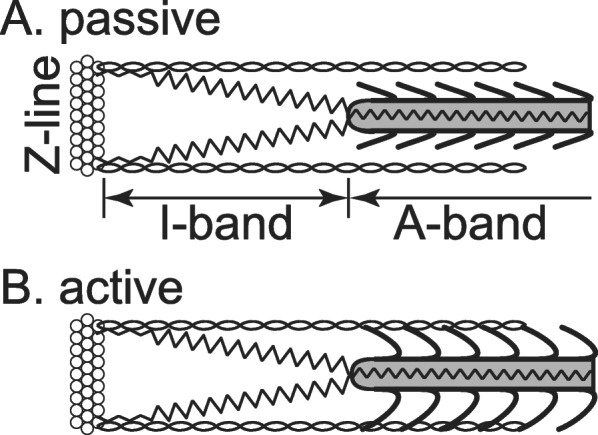
Titin. Schematic illustration of titin in the passive state of a half-sarcomere, assuming no cross-bridge connections between actin and myosin. For these idealized conditions, titin filaments can be considered mechanically in series with the myosin filament, while in the active state, with cross-bridge formation between actin and myosin, titin filaments are not in series with the myosin filament, but behave like a parallel element to the cross-bridges; that is, titin forces add algebraically to the cross-bridge forces to give the entire force in an isolated half-sarcomere

The molecular spring titin is interesting to contemplate as a series elastic element. Titin spans the half-sarcomere from the M-line to the Z-band. It is thought to be rigidly attached to the myosin filament with no (or only very little) possibility for elongation in the A-band region of the sarcomere (Fig. 5). However, titin runs freely across the I-band region from the end of the myosin filament to approximately 50 nm away from the Z-band where it combines with the actin filament [32–34]. The I-band region of titin is known to be extensible, and is thought to be virtually elastic if elongation is small and no immunoglobulin domains of titin are unfolded [35, 36]. Because of this structural arrangement, titin filaments are in series with the myosin filament in the passive muscle, assuming the idealized case that there are no (cross-bridge) connections between actin and myosin in the passive state. In the active state, when cross-bridges are formed between actin and myosin, titin is not in series with the myosin filament anymore, as its force would not represent the force carried by the myosin filament, while in the idealized passive state, it would. In the active state, titin acts more like a spring that is in parallel to the cross-bridges; that is, its force adds algebraically with the forces of the cross-bridges interacting between an actin-myosin pair. Note, that in normal muscle, each half-myosin is associated with six titin filaments [37], so when attempting to calculate the forces in a titin filament in a passive muscle, this ratio needs to be kept in mind. Furthermore, in disuse atrophied muscles, or in spastic muscles of children with cerebral palsy, this 6:1 ratio of titin filaments vs. half myosin, becomes smaller and might be as low as 3:1 [38, 39]. However, like for the cross-bridge elasticity, titin elasticity is not in series with the entire muscle.

A single myofibril consists of sarcomeres arranged in series with one another. That is, each sarcomere transmits the same force at any given time as the next one. Therefore, the instantaneous forces measured at the end of a myofibril are the same as the instantaneous forces transmitted by each sarcomere in that myofibril (which is the primary reason why single myofibril mechanical experiments are so powerful). Similarly, the Z-band in a single myofibril is in series with its neighbouring sarcomeres, and any force transmitted across the Z-band will be the same as the force of the sarcomeres. However, multiple myofibrils in a muscle/fibre are structurally arranged in parallel, and sarcomeres of neighbouring myofibrils are connected by various structural proteins (desmin being the most acknowledged), and thus, the Z-bands in neighbouring myofibrils and fibres are not in series with each other. As a consequence of this highly connected and integrated arrangement of sarcomeres in myofibrils and fibres, the system of sarcomeres is mathematically redundant, and it is impossible to determine the force in a given sarcomere of a muscle, even when the muscle force and the target sarcomere length are known.

#### Tendon and aponeuroses

**Fig. 6:**
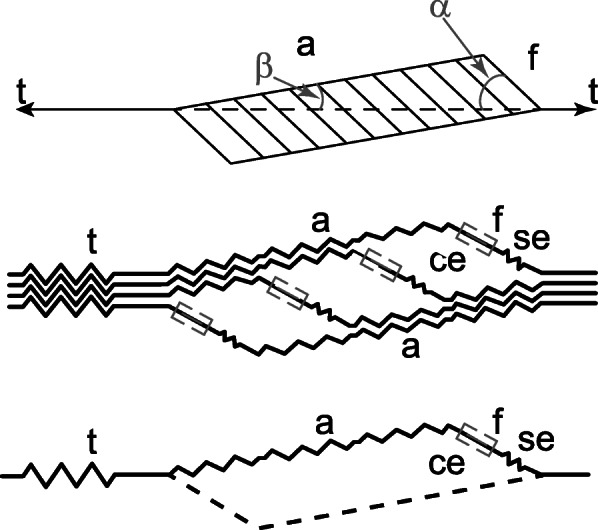
Unipennate muscle. Schematic illustration of a unipennate muscle (top panel), and associated structural elements. t = tendon, a = aponeurosis, f = fibre, ce = contractile element, se = series elastic element. As shown in the middle and bottom panel, Ettema and Huijing(1990) [40] assumed that a fibre and associated contractile element were in series with the aponeurosis and associated tendon. This idea will reappear below. [Reprinted with permission from New York: Springer Verlag, Multiple Muscle Systems, Ettema and Huijing 1990 [40]]

Returning to the discussion of series elasticity in entire muscles, tendons and aponeuroses have often been treated, implicitly or explicitly, as the series elastic elements of skeletal muscles. The argument frequently made is that since tendon and aponeurosis are structurally in series with the muscle fibres, as suggested in the schematic drawing by Ettema and Huijing (1990) [40] (Fig. 6), they are also mechanically in series. This thinking is exemplified by measurements of aponeuroses and tendon elongations, relating these elongations to muscle force, and then assuming that there is a relationship between muscle force and tendon/aponeurosis length that is governed solely by the constitutive equation of the aponeurotic/tendinous tissue. While this thinking is justified for the free tendon of a muscle [14, 23, 41], it is not for the internal aponeuroses of muscles, as has frequently been done [e.g .[42, 43]].

Implicitly, aponeuroses tissues have been assumed to be series elastic elements of muscles in studies where “series elasticity” is defined/obtained by subtracting fibre/fascicle length from the entire muscle-tendon unit length (e.g. [17, 21]. It has been shown theoretically that forces in aponeuroses are not the same as in the free muscle tendon [e.g.43], and that the pressure and shear rigidity of muscles play a crucial role in the relationship between tendon and aponeurosis forces [e.g .[24, 44]]. However, before conducting a detailed theoretical analysis of the relationship between tendon and aponeuroses forces, and sharing experimental observations of directly measured muscle forces and aponeurosis deformations, we would like to define what we mean by the (free) tendon and (inner) aponeuroses of muscles.

**Fig. 7:**
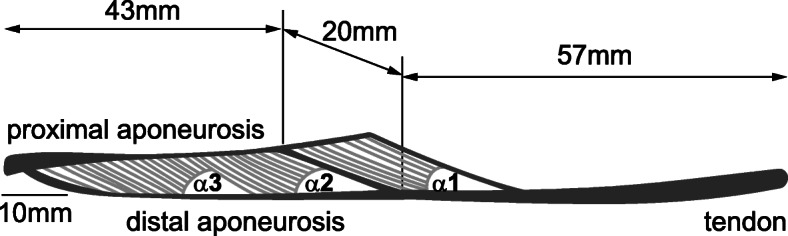
Tendon and Aponeuroses. Midsagittal, scaled section of a cat medial gastrocnemius muscle with approximate dimensions indicated. The free tendon (hereafter simply referred to as tendon) is the connective tissue external to the muscle. The lateral or distal aponeurosis is an extension of the distal external tendon, reaching into the muscle, and fibres are attaching to it. The medial or proximal aponeurosis is a continuation of the short, proximal tendon of the muscle, and fibres insert into it. The force in the tendon always reflects the total (active and passive forces) produced by the muscle. Tendon force is constant along its length. The forces in the aponeuroses do not depend in a simple manner on the muscle force, but depend crucially on the instantaneous shear modulus and pressure of the muscle, and vary along the aponeuroses, with forces in the aponeuroses greatest towards their tendinous insertions and decreasing along the aponeurosis towards the interior of the muscle

For simplicity, but without loss of generality, let us assume we are dealing with a unipennate muscle, for example, the cat medial gastrocnemius muscle (Fig. 7). The free tendon of the muscle is defined as the connective tissue, tendinous material that is external to the muscle belly, as indicated in Fig. (7). The cat medial gastrocnemius has two aponeuroses, one located proximally and the other distally on the muscle (Fig. 7). They are composed of connective tissues to which the muscle fibres insert. The aponeuroses, by virtue of their location, are exposed to the pressure and shear forces exerted by the muscle upon contraction, while the tendon is not. Pressure and shear forces need to be considered when calculating the forces transmitted by aponeuroses, while the tendon simply transmits whatever force is produced by the muscle’s contractile and passive structures [e.g.23]. Therefore, the tendon can safely be considered mechanically “in series” with the muscle, while the aponeuroses cannot.

Although illustrated on the example of a unipennate muscle, the general statement that the free tendon is always mechanically in series with the contractile part of the muscle, the muscle belly, is correct in general for fusiform and multi-pennate muscles. Similarly, aponeuroses, as defined above for a unipennate muscle, are never mechanically in series with the free tendon or the contractile part of the muscle, and in contrast to the free tendon, will always have a force that varies along its length. Again, this statement is generally correct for any muscle that has aponeuroses embedded within the contractile part of the muscle.

Aponeuroses are sometimes also referred to as the pearly white fibrous tissues that take the place of tendons in flat muscles having a wide area of attachment. For muscles with such wide areas of attachment, for example in the human abdominal area, the hand and feet, aponeuroses may lie outside the muscles and may be arranged in series with the contractile elements of muscles. However, for the sake of clarity (and also for its common use in biomechanics research), we consider aponeuroses here as shown in Fig. (7); that is, aponeuroses are internal to the muscle with the contractile fibres inserting into them.

#### Why aponeuroses cannot be considered “in series” with either the free tendon or the muscle: theoretical considerations

**Fig. 8:**
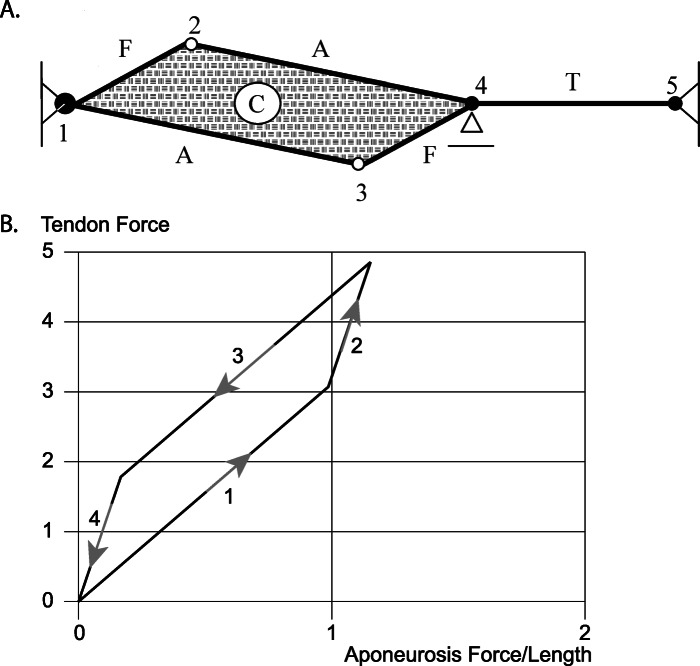
Series Elasticity. Schematic representation of a unipennate muscle (**a**) with contractile fibres F, an elastic tendon T, elastic aponeuroses A, and an elastic, incompressible material C that enforces iso-volumetricity (i.e. constant area in this example) during muscle contraction. (**b**) The relationship between muscle (tendon-) force and aponeurosis force (which is equivalent to aponeurosis length since the aponeurosis is assumed to be linearly elastic) is shown for a muscle that is initially passively stretched (1), then activated while kept at a constant length (2), then shortened in the activated state (3), and finally deactivated to return to its passive state at the original length (4). Note that the aponeurosis force is always smaller than the corresponding muscle (tendon-) force. Note further that muscle (tendon-) force and aponeurosis forces are not uniquely related, and that plotting muscle (tendon-) force in this manner against the aponeurosis force results in a counter-clockwise loop. If one assumed (as has sometimes been done) that the aponeurosis is in series with the tendon, one would obtain positive net mechanical energy from the (purely elastic) aponeurosis for this stretch shortening cycle starting and ending with zero muscle (tendon-) force. Such energy creation of an elastic material is not possible (it violates the laws of thermodynamics), and thus proves that such an interpretation (i.e. assuming that for this example the aponeurosis is in series with the muscle/tendon) is not correct. [Reprinted with permission from Elsevier Science Publishers, Journal of Biomechanics, Epstein et al. 2006 [24]]

Let us assume we have a muscle with contractile fibres, purely elastic aponeuroses (A), and a purely elastic tendon (T) (Fig. 8a) [25]. We further assume that the muscle is incompressible. Incompressibility is enforced by an incompressible, elastic material (C) inside the borders formed by the aponeuroses and the contractile fibres. For this simple representation of a muscle, we can calculate the forces in T and A at any time for an assumed contraction/force of the fibres. Let us further assume we stretch the muscle first passively until a certain amount of passive force is developed, then activate the muscle isometrically, shorten it back to its original length while activated, and finally deactivate the muscle, so it has reached its initial passive configuration (Fig. 8b). When going through this dynamic contraction, the forces in the aponeurosis are always smaller than in the tendon, and the aponeurosis forces change when the elasticity, specifically the shear modulus of the incompressible muscle (C – Fig. 8a), is changed (not shown). When assuming the shear modulus to be zero (which is unrealistic for muscle tissue), the hysteresis observed in Fig. (8b) for the stretch-shortening cycle disappears (not shown). But even for this extreme case, the tendon and aponeurosis forces are not the same [24]. Furthermore, the result obtained here is not exclusive to an incompressible muscle, but would also be obtained with a compressible material.

The theoretical example discussed above has been taken from one of our previous publications, and details of the calculations and the model can be obtained from [24]. We conclude that for this representation of a unipennate muscle, the force in the aponeurosis is not related in a simple way to the force in the tendon; i.e., the muscle force. Even though in the example we only discuss conditions of muscle activation and deactivation, and an isometric and concentric contraction, the findings are independent of the contractile conditions and are also correct for an eccentric contraction or a stretch-shortening cycle.

**Fig. 9:**
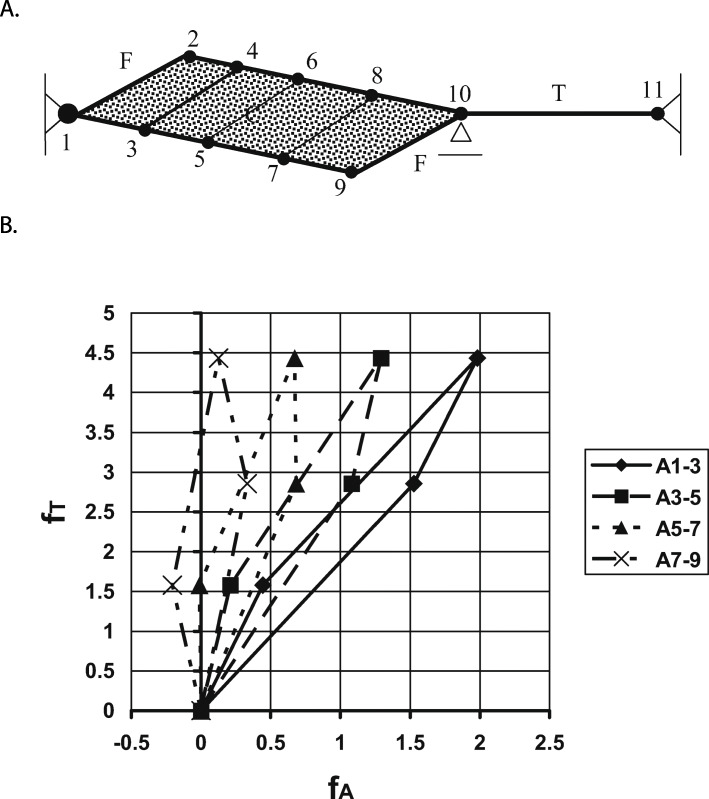
Series elasticity. **a** Schematic representation of a muscle with contractile fibres (F), elastic tendon (T), eight elastic aponeurosis segments (labelled from 1 to 10), and an elastic, incompressible material (C). This multi-panelled muscle is subjected to the same stretch-shortening cycle as described in Fig. (8). **b** The corresponding tendon forces (f_T_) as a function of the aponeuroses forces (f_A_), where the aponeurosis forces are equivalent to aponeuroses lengths because of the assumed linear elasticity of the aponeurosis segments. Note that the aponeurosis forces are always smaller than the corresponding tendon forces, that the aponeurosis forces do not relate to the tendon forces in a simple and unique manner, and that the aponeurosis forces (and thus the aponeurosis lengths) can be negative for some conditions where the tendon forces are substantial. A negative aponeurosis force is likely not possible in a real muscle (as aponeuroses would fold/buckle and not resist compressive forces). However, aponeuroses shortening upon muscle activation and increasing muscle forces has been observed experimentally as described in the text. [Reprinted with permission from Elsevier Science Publishers, Journal of Biomechanics, Epstein et al. 2006 [24]]

In a further refinement of the model shown above, we can divide the muscle into multiple panels separated by contractile fibres (Fig. 9a), and repeat the stretch-shortening cycle from the previous example. When doing so, it can be shown that the aponeurosis force becomes smaller when going from the “attached” end (panel 1–3) to the “free” end (panel 7–9 – Fig. 9b – bottom aponeurosis). This result is consistent with the observed “thinning” of aponeuroses from the “attached” to the “free” end, as for example illustrated in the medial gastrocnemius of the cat (i.e. a thinning of the medial aponeurosis from the left “attached” to the right “free” end – Fig. 7). Furthermore, observe that the aponeurosis forces can be negative (corresponding to a shortening of the aponeurosis) in the presence of positive tendon forces (Fig. 9b – panel 7–9). A shortening of aponeurosis segments upon muscle activation, and associated increase in force, has been observed experimentally [44,45etc.]. For the details of this previously published analysis, please refer to Epstein et al. [24].

We conclude from these theoretical considerations that aponeuroses forces are not the same as tendon forces, that aponeuroses forces are not in a constant ratio to tendon forces, that aponeuroses forces are smaller than tendon forces, and that they can be negative (aponeuroses shortening) in the presence of positive tendon forces. Furthermore, aponeuroses forces vary along the aponeuroses and tend to be greater at the “attached” compared to the “free” end, in agreement with the generally observed tapering of the thickness of aponeuroses from the “attached” to the “free” end.

#### Why aponeuroses cannot be considered “in series” with either the free tendon or the muscle: experimental observations

Even though the muscle models developed above contain the essential elements of a real muscle: contractile fibres, “elastic” aponeuroses, an incompressible muscle substance, and an “elastic” tendon, its predictions might not reflect a real muscle. In particular, one might argue that there is no direct measurement of aponeurosis forces, and indeed, to our best knowledge, such forces have never been measured in an intact muscle. However, when measuring aponeuroses elongations for a variety of conditions, observations have been made that are incompatible with an “in series” arrangement of aponeuroses with either tendons or with muscle fibres.

**Fig. 10:**
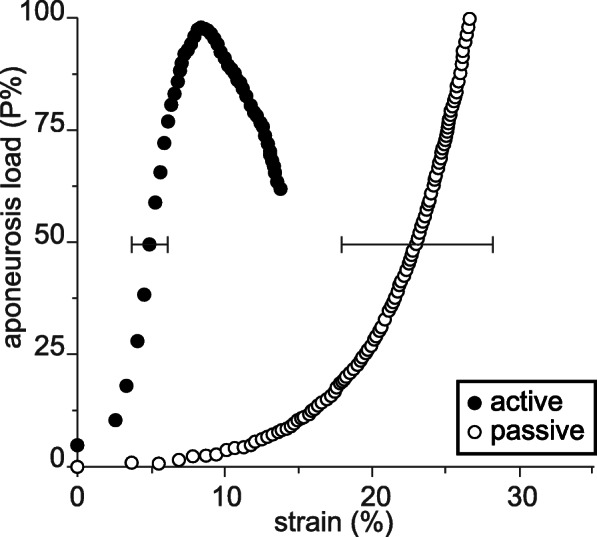
Aponeurosis mechanics. Aponeurosis load as a function of aponeurosis strain for active and passive conditions in frog semitendinosus muscles. The observation made here that aponeurosis strains were significantly smaller for the active compared to the passive muscle is inherently correct and agrees with the theoretical considerations made above and experimental observation made by others [24, 46]. However, this graph (reproduced in its original form) must be considered and interpreted with caution, because the variable on the vertical axis is not, as indicated, the aponeurosis load, but it is the load on the tendon. Since tendon loads and aponeurosis loads are not related in a simple or unique manner, and differ substantially between corresponding (same tendon force) active and passive conditions, the figure, as depicted by Lieber et al. [45] has led to misinterpretations of the aponeurosis mechanics that were at play in this experiment [Reprinted with permission from Karger, Cells Tissues Organs, Lieber et al. 2000 [45]]

For example, Lieber et al. [45] measured aponeuroses elongations as a function of tendon force in frog semitendinosus for passive and active muscle conditions. They found that aponeurosis elongations were significantly greater in the passive compared to the active muscle (Fig. 10). At corresponding force levels (50% of the maximal isometric force at optimal length), they found aponeurosis strains of about 5 and 23% for the active and passive conditions, respectively (Fig. 10). They concluded from this result that an *“active contraction actually altered aponeurosis material properties”*. It seems unlikely that a non-contractile material, like the aponeurosis of the frog semitendinosus muscle, could change its material properties upon muscle activation. Rather, one would suspect that the material properties of the aponeurosis remained the same but the forces acting on the aponeurosis, for a given muscle force, differ between the active and passive conditions, and were not related in a simple way to the tendon force. The error made in the interpretation by Lieber et al. (2000) was that they assumed that the tendon force, which they measured directly, was the same as the aponeuroses force, independent of the muscle length and independent of the muscle’s active state. Activation in muscles is associated with increases in internal pressure and changes in stiffness, including shear stiffness [23, 24, 44, 47, 48], thus assuming that the tendon force is equivalent to the aponeurosis force, and implying material properties based on such thinking, will lead to erroneous interpretations of aponeurosis function, mechanical properties, and energetic results. The experimental observations by Lieber et al. [45] are captured generically in our theoretical model above (Fig. 9b), where the relationship between tendon force and aponeurosis force changes when the muscle is activated, and an increase in tendon force with activation was associated with a decrease in aponeurosis force and aponeurosis length, agreeing with the experimental observations by Lieber et al. [45].

Significantly shorter aponeurosis length in active compared to passive muscle have been published prior to the Lieber et al. [45] paper. For example, Zuurbier et al. [46] reported that *“aponeurosis length as a function of aponeurosis force was significantly shorter in the active compared to the passive … condition”*, for the proximal aponeurosis of the unipennate medial gastrocnemius muscle of the rat. This statement reflects their observation of aponeurosis length in active and passive muscle for corresponding muscle forces, and they relate (again erroneously) the tendon force to the aponeurosis force, not accounting for the fact that the relationship between tendon and aponeurosis force changes with activation due to the increase in muscle pressure and shear stiffness upon muscle activation. This does not diminish their observation, merely the interpretation of their results, as activation of a muscle, and associated increase in tendon force, can lead to decreased aponeuroses forces, as shown in our theoretical considerations above (Fig. 9b).

Magnusson et al. [42] were among the first to claim that they quantified the mechanical properties of aponeuroses in intact human skeletal muscles. Their highly cited paper represents a careful attempt of quantifying the stiffness and Young’s modulus of human medial gastrocnemius tendon and aponeurosis. However, they estimated the aponeurosis force “*…*. *by dividing the externally measured moment by the tendon moment arm.”* While this is perfectly acceptable for tendon/muscle force estimates, this approach is not appropriate for estimating the variable forces in the aponeurosis, as it (typically vastly) overestimates the aponeurosis forces. They found similar elongations for the proximal and distal segments of the medial gastrocnemius aponeurosis and concluded that “…. *the stiffness was similar for the two regions.”* Their conclusion (again) is based on the assumption that equal elongation (of the distal and proximal aponeurosis segments) was associated with equal forces acting on these two segments, which is incorrect as the aponeurosis forces vary along the aponeurosis (and thus are likely substantially different for the distal and proximal segments), and the aponeurosis forces are not equivalent to the muscle/tendon force. Their calculation of aponeurosis stiffness, thus, is an (likely vast) overestimation of the true value, which is confirmed in studies where the true (isolated) aponeurosis material properties have been compared to the aponeurosis elongations and equivalent tendon forces in intact muscles [46]. Furthermore, their conclusion that proximal and distal aponeurosis stiffness are the same, is probably not correct. Rather, the similar elongations of these two segments likely reflects a continuous change in the stiffness of the aponeurosis along its length that matches the changing in vivo forces acting along the aponeurosis in such a manner that aponeurosis strains are “constant” along its length.

In the above examples, material properties of intact aponeurosis have been implied from the elongations of the aponeuroses and the corresponding forces in the muscles/tendons. The implicit assumption in these examples is that material properties, such as stiffness or the Young’s modulus, can be derived by assuming that the forces acting on the aponeuroses are those measured at the distal end of tendons. From a mechanical point of view this is incorrect, as shown in the theoretical considerations above. It leads (typically) to overestimations of the actual aponeurosis stiffness.

Aside from ill-fated attempts to measure the material properties of aponeurosis in intact human skeletal muscles [e.g.41], another frequently used mechanical concept is that of the storage and release of mechanical energy in muscle series elastic elements. This topic will be discussed in the following paragraphs.

#### Storage and release of energy in “series elastic” muscle elements

Many movements in animals, including humans, are cyclic in nature and are associated with a stretch-shortening cycle of the muscle-tendon unit complex [49]. It has been argued that many muscles are built to take mechanical and energetic advantage of the stretch-shortening cycle through their series elastic elements (i) by affecting the rate of change in the contractile elements of the muscle [1], (ii) by storing and releasing potential energy in the series elastic elements [20]; and (iii) by increasing force/work in the shortening phase of the stretch-shortening cycle through mechanisms of residual force enhancement [18, 22].

Muscle series elasticity, in this context, has frequently been defined, implicitly or explicitly, as the elements *“obtained by subtracting muscle fiber length from origin to insertion distance”* [e.g.20]. This definition has been applied to measure elastic energy storage and release in intact muscles of freely moving animals. For example, Roberts et al. [17] calculated the tendon energy recovery in the lateral gastrocnemius of Turkeys using the muscle force (measured at the calcified tendon) and the tendon/aponeurosis stiffness (calculated by the elongation of tendon and aponeurosis in isometric contractions and assuming muscle/tendon force to be equivalent to the variable forces acting along the aponeurosis). This procedure leads to overestimates of the actual aponeurosis stiffness as muscle pressure and shear forces created upon muscle activation are neglected, resulting in overestimations of the energy recovered by the aponeurosis.

**Fig. 11:**
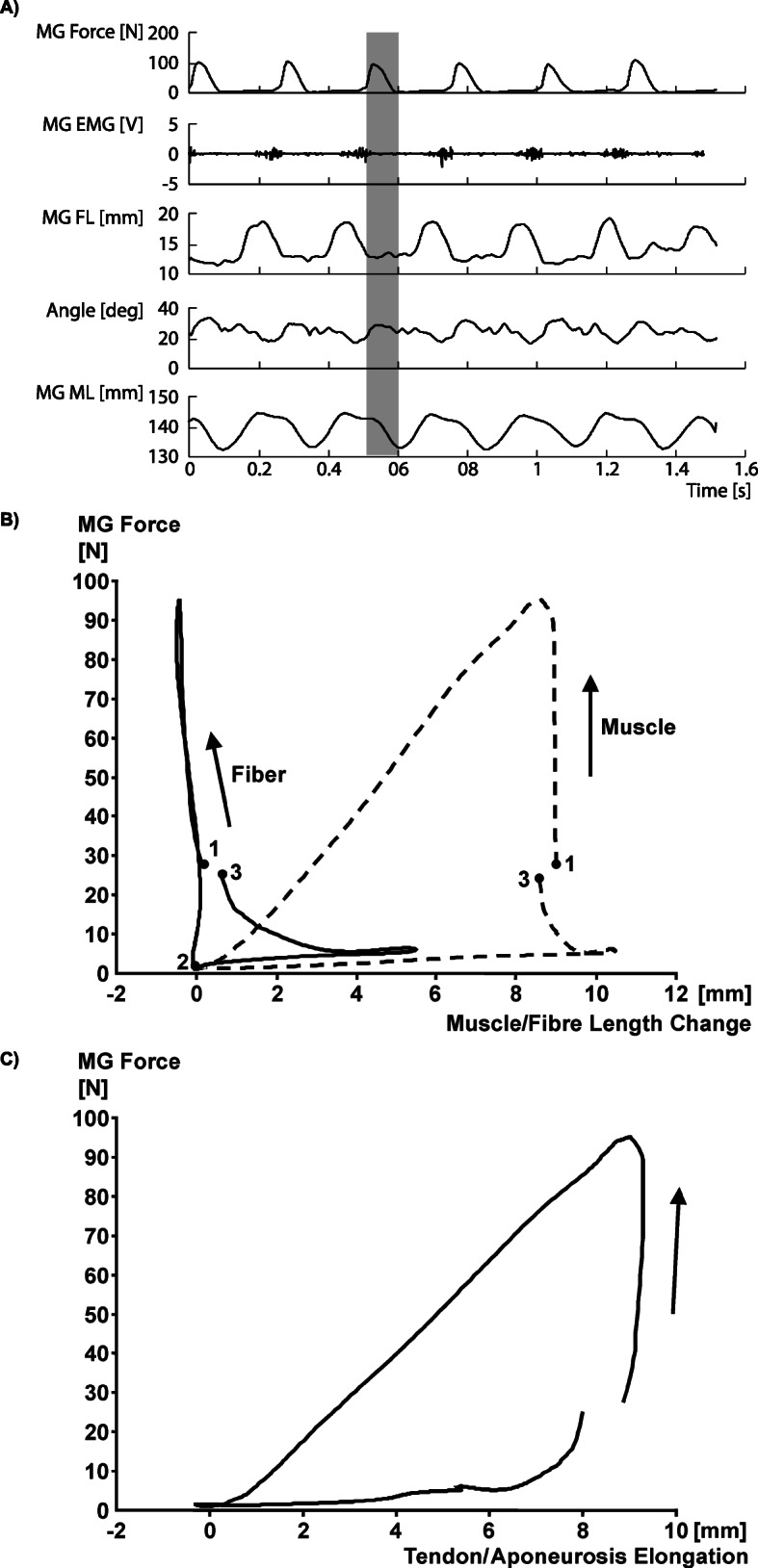
In vivo muscle mechanics: (**a**) Cat medial gastrocnemius forces, electromyographical signals, fascicle lengths, angle of pennation, and whole muscle tendon unit length as a function of time for a cat galloping at 4.0 m/s. Muscle forces were measured directly using buckle tendon force transducers [50, 51], and muscle lengths, fascicle lengths and angles of pennation were measured directly using four sonomicrometry crystals that were attached to the end of mid-sagittal plane fascicles identified by micro-stimulation [52]. **b** Average muscle length and fascicle length for five consecutive step cycles. **c** Average tendon/aponeurosis elongation (obtained by subtracting fascicle length from total muscle-tendon unit length) vs. muscle force (measured at the distal end of the tendon using a buckle type force transducer) from the step cycles shown in (**a**) and depicted in (**b**). Note that the tendon/aponeurosis length vs. muscle force describe a counter-clockwise loop. If one assumed that the aponeurosis was in series with the tendon, and thus had the same instantaneous force as measured at the tendon, one would need to conclude that the aponeurosis produces positive work. However, since aponeuroses are passive, (visco-) elastic structures, they absorb energy; they cannot create energy, thereby proving that the aponeurosis is not mechanically in series with the tendon (and the tendon force)

**Fig. 12:**
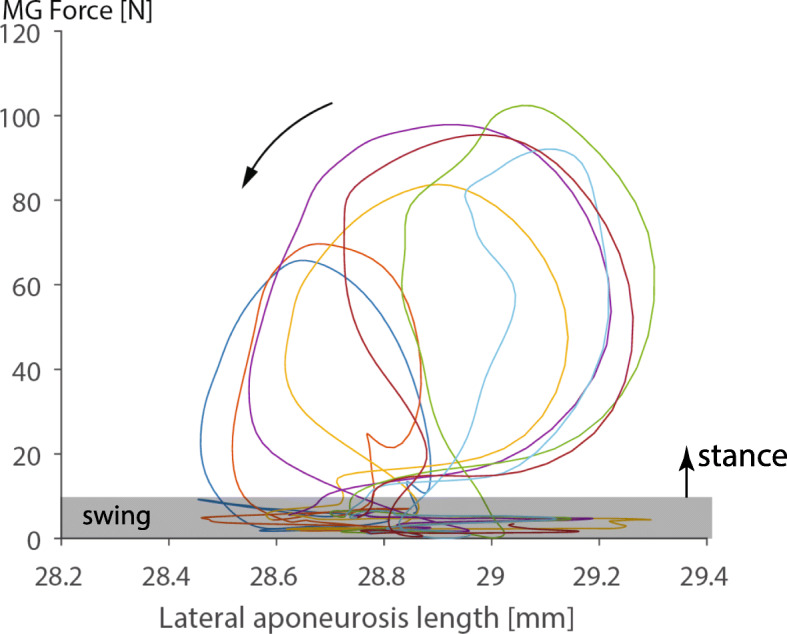
Aponeurosis elongations. Medial Gastrocnemius (MG) muscle force vs. lateral aponeurosis segment length changes for six consecutive step cycles of a cat galloping at 4 m/s. Segmental aponeurosis length changes occurring at force levels less than about 10 N correspond to the swing phase, while the open, counter-clockwise loops above about 10 N correspond to the stance phase of running. Note that the excursions for the swing phase (low forces) and stance phase of the step cycles (high forces) are about the same. Furthermore, note that the loops formed during the stance phase are in a counter-clockwise direction. If we assumed (incorrectly) that the muscle force (measured at the distal end of the tendon) was in series with the lateral aponeurosis segment depicted here, we would conclude (incorrectly) that the aponeurosis produces energy during each step cycle. Therefore, we can safely conclude that the aponeurosis is not related in a simple (in series) way to the tendon force. In other words, the tendon force does not reflect the force acting on this particular segment of the aponeurosis, and since we do not know the instantaneous and location-dependent force on the aponeurosis, we cannot (easily) determine what the aponeurosis material properties are, nor can we (easily) estimate what energy might be stored and released in the aponeurosis segment during these step cycles. At best, we might be able to estimate the aponeurosis material properties and energy contributions using a refined version of the model shown in Fig. (8)

We measured the force, muscle-tendon unit length, and a mid-belly fascicle length in the cat medial gastrocnemius muscle for a variety of locomotor conditions, including walking, trotting, galloping, and jumping (Fig. 11a). In analogy with van Ingen Schenau et al. [21] and Roberts et al. [17], we then subtracted the instantaneous fascicle lengths from the instantaneous muscle tendon unit length (Fig. 11b), and plotted this difference (assumed to represent the series elastic element of muscle) against the muscle force measured at the distal end of the gastrocnemius tendon (Fig. 11c). When doing this, we consistently observed a positive work loop for the assumed series elasticity. However, since a (visco-) elastic element can at best release the same amount of energy that was initially stored in it, and thus cannot create a positive work loop as shown in Fig. (11C), we must conclude that the muscle/tendon force measured is not related in a direct and simplistic manner to the aponeurosis elements of the muscle. In other words, subtracting the fascicle length from the total muscle-tendon unit length (and accounting for the angle of pennation) does not provide a series elastic element in the mechanical sense [53]. The forces acting on the aponeurosis are not related in a simple manner to the muscle/tendon forces. Assuming that they are can give results of work/energy production that are thermodynamically not possible. In order to demonstrate that aponeurosis elongations are not related to muscle/tendon force, we also measured segmental elongations of the lateral aponeurosis of the cat medial gastrocnemius muscle for multiple step cycles and various locomotor conditions. In all cases, the segmental aponeurosis elongations were not related to the muscle/tendon force in a unique manner (Fig. 12). Rather, the range of aponeurosis elongations was similar for the recovery phase of the step cycle (where muscle forces were small), and the active force producing stance phase (where forces were high). Note also that all “force-elongation loops” for the stance phase of locomotion in this example are counter-clockwise, that is, if we assumed that the aponeurosis was “in series” with the muscle/tendon (where the force was measured), we would obtain positive work loops, once more illustrating that the aponeurosis length changes cannot be related directly to the tendon force by assuming a mechanical “in series” arrangement.

## Conclusion

Tendon, aponeuroses, and muscle fibres are related mechanically in a complex and non-intuitive manner. Assuming these elements to be in series with each other, as has been done in the calculation of material properties of aponeuroses in intact muscles, and in the calculation of storage and release of elastic energy in muscles, has led to results that are thermodynamically not possible. We conclude, based on published evidence by others [45, 46], theoretical considerations [24, 44], and our own experimental results, that aponeuroses (as defined here) are not mechanically in series with tendons or muscle fibres, and should not be treated as such. These elements may well be structurally in series with each other, but when associating mechanical terms to “series elastic” elements of muscles, such as stiffness, Young’s modulus, or storage and release of mechanical energy, we need to be careful to relate the appropriate forces to the appropriate structures. This has often been ignored in the past, leading to confusion about material properties of tendons and aponeuroses, and the energetics of muscle contraction.

## Data Availability

All materials of this review article that are from the author’s own research are made available to anybody upon request.

## Abbreviations

A-band: anisotropic (dark-) band in the sarcomere
CE: Contractile Element
f: fibre
*F*: Force
*F*_*cb*_: Cross bridge force
I-bands: isotropic (light-) band in the sarcomere
*k*: Spring constant
*k*_*cb*_: Cross-bridge spring constant
MG: Medial Gastrocnemius
M-line: centre or midline of the sarcomere
SE: Series Elastic Element
*x*: elongation
Z-band: end line or “Zwischenscheibe” of the sarcomere
